# Diurnal Concentration of Urinary Nitrogen and Rumen Ammonia Are Modified by Timing and Mass of Herbage Allocation

**DOI:** 10.3390/ani9110961

**Published:** 2019-11-13

**Authors:** Ignacio E. Beltran, Pablo Gregorini, José Daza, Oscar A. Balocchi, Alvaro Morales, Ruben G. Pulido

**Affiliations:** 1 Graduate School, Faculty of Veterinary Sciences, Universidad Austral de Chile, PO Box 567, Valdivia, Chile; Ignacio.beltran.gonzalez@gmail.com; 2 Institute for Agricultural Research, Remehue Research Centre, PO Box 24-0, Osorno, Chile; 3 Department of Agricultural Science, Lincoln University, PO Box 7647, Lincoln, New Zealand; pablo.gregorini@lincoln.ac.nz; 4 Animal Production Institute, Faculty of Agricultural Sciences, Universidad Austral de Chile, PO Box 567, Valdivia, Chile; jose_daza11@hotmail.com (J.D.); obalocch@uach.cl (O.A.B.); 5 Animal Science Institute, Faculty of Veterinary Sciences, Universidad Austral de Chile, PO Box 567, Valdivia, Chile; alvaro.morales@uach.cl

**Keywords:** circadian nitrogen excretion, grazing management, rumen ammonia, dairy cows

## Abstract

**Simple Summary:**

Low nitrogen use efficiency in grazing dairy cows leads to high urinary N excretion, which contributes to greenhouse gases emission. This problem has been associated with high N and low energy intake, increasing rumen ammonia (NH_3_) concentration, and thereby, increasing urinary N excretion. Under this situation, it is important to discover nutritional and grazing management strategies that allow reduced urine N excretion in the pasture. This study evaluated whether changes in time of herbage allocation and herbage mass modify the diurnal pattern of urinary nitrogen (N) concentration and ruminal NH_3_ of lactating dairy cows. We found that the combination of time of herbage allocation and herbage mass modified rumen NH_3_ production and urinary N concentration. Results suggest that maintaining cows in the holding pen at the milking parlor for two hours after morning and afternoon milking could allow collection of urine from cows in the slurry pit during peak N concentration, returning cows to the pasture at a time of day when urinary N concentration is decreased.

**Abstract:**

The objective of this work was to evaluate whether changes in time of herbage allocation and herbage mass (HM) (low (L) or medium (M)) modify the diurnal pattern of urinary nitrogen (N) concentration and ruminal ammonia (NH_3_) of lactating dairy cows. Four Holstein-Friesian cows fitted with rumen cannula were randomly allocated to one of four treatments: 1) low herbage mass in the morning (L-AM) (Access to new herbage allocation after morning milking with a herbage mass (HM) of 2000 kg DM/ha); 2) low herbage mass in the afternoon (L-PM) (Access to new herbage allocation after afternoon milking with a HM of 2000 kg DM/ha); 3) medium herbage mass in the morning (M-AM) (Access to new herbage allocation after morning milking with a HM of 3000 kg DM/ha); and 4) medium herbage mass in the afternoon (M-PM) (Access to new herbage allocation after afternoon milking with a HM of 3000 kg DM/ha). A four by four Latin Square design with four treatments, four cows, and four experimental periods was used to evaluate treatment effects. Rumen NH_3_ concentration was greater for L-AM compared to L-PM and M-PM at 13:00 and 16:00 h. Urine urea and N concentrations were lower for M-AM compared to L-AM. Urine N concentration was greater for L-AM than other treatments at 10:00 hours and greater for M-PM compared to M-AM at 16:00 hours. Results suggest that maintaining the cows in the holding pen at the milking parlor for two hours after morning grass silage supplementation for L-AM and for two hours after afternoon grass silage supplementation for M-PM, could allow collection of urine from cows at the holding pen and storage of urine in the slurry pit during the time of peak N concentration, returning cows to the pasture at a time of day when urinary N concentration is decreased.

## 1. Introduction

The environmental effect of livestock has increased the need to develop environmentally friendly strategies for pasture-based animal production systems. Ruminants are characterized as being much less efficient at utilizing high quality dietary proteins than non-ruminants [1], with grazing dairy cows having a nitrogen use efficiency (NUE) ranging between 13–31% [2,3], which indicates that a high amount of nitrogen (N) intake is excreted through the urine and feces. Urinary nitrogen (UN) excretion via ammonia (NH_3_) volatilization, nitrate leaching, and dissipation of N as nitrous oxide, nitric oxide, and nitrogen dioxide [4,5] is commonly associated with environmental pollution from farming.

The high UN excretion in grazing dairy cows has been associated with a limited supply of energy from temperate herbage [6]. Therefore, the supply of amino acids often exceeds animal requirements [7] and when rumen energy is limited, amino acids are deaminated and excreted by ruminal bacteria as NH_3_, which is converted to urea by the liver and then excreted via urine [8,9].

Increments in aboveground herbage mass (HM) reduce crude protein (CP) content while increasing the proportion of water-soluble (structural and non-structural) carbohydrates (WSC) [10]. On the other hand, timing of herbage allocation, e.g. AM versus PM, can be used to modify herbage intake, by increasing dry matter (DM) and WSC content and diluting CP content of herbage due to changes in grazing behavior, photosynthetic activity and moisture lost [11,12]. Thus, combining HM and timing of herbage allocation could be used as a strategy to modify the WSC/CP ratio in herbage and thereby diurnal UN excretion as a result of changes in N intake and rumen NH_3_ concentration. 

In pasture-based dairy systems over 81% of N is excreted in the paddocks, indicative that lower than 20% of urine N excreted is collected in the milking parlor [13]. Understanding the daily pattern of UN excretion and how to fit it with the permanence of cows in the pasture or facilities, a potential strategy to reduce the urine N excreted into the pasture could be a combination between animal nutrition strategies and modification in the animals’ management during the day. The objective of this study was to evaluate whether changes in timing of herbage allocation and herbage mass (low (L) or medium (M) modify the daily pattern of UN concentration, rumen NH_3_ and grazing behavior of lactating dairy cows.

## 2. Materials and Methods

All procedures in this experiment were approved by the Animal Welfare Committee of Universidad Austral de Chile (grant number 255/2016).

The experiment was carried out from May 5th to July 1st, 2016 at the Agricultural Research Station at Austral University of Chile (latitude 39 ° 47 ’S and longitude 73 ° l4’ W, annual rainfall 2500 mm). 

### 2.1. Cows, Experimental Design and Treatments

Four Holstein-Friesian cows fitted with rumen cannula were selected according to milk production (24.7 ± 2.8 kg/d), body weight (BW) (580.6 ± 51.7 kg), days in milk (DIM) (74 ± 17.1), and body condition score (BCS) (3.1 ± 0.3; one–five point scale) [14] and randomly allocated to one of four treatments: 1) low herbage mass in the morning (L-AM) (Access to new herbage allocation after morning milking with a HM of 2000 kg DM/ha); 2) low herbage mass in the afternoon (L-PM) (Access to new herbage allocation after afternoon milking with a HM of 2000 kg DM/ha); 3) medium herbage mass in the morning (M-AM) (Access to new herbage allocation after morning milking with a HM of 3000 kg DM/ha); and 4) medium herbage mass in the afternoon (M-PM) (Access to new herbage allocation after afternoon milking with a HM of 3000 kg DM/ha). Experimental cows in each treatment grazed with 10 other cows which allowed expression of normal gregarious behavior instead of physiological and behavioral stress responses elicited by isolation [15]. 

A four by four Latin Square design with four treatments, four cows, and four experimental periods was used for the present study. Each experimental period had a 14-d duration, where the first 13-d of each period was a diet adaptation time and the last day (day 14) where samples were collected. At the end of each experimental period the cows were reassigned to a different treatment group.

All groups were strip-grazed paddocks with an aboveground herbage allowance of 21 kg DM/cow/d offered at 09:00 for L-AM and M-AM treatments and at 16:00 hours for L-PM and M-PM treatments. In addition, all cows were supplemented with 3.5 kg DM/d of concentrate which was fed in two equal amounts during milking (07:00 and 14:00 hours). The concentrate was comprised (% of DM basis) of 49.3 corn, 11.5 soybean meal, 30.0 beet pulp, 4.6 beet molasses, and 4.5 mineral mix. All cows received 3.0 kg DM/d of grass silage which was fed in two equal amounts after milking. 

### 2.2. Herbage and Grazing Management

All cows grazed a 20-ha perennial ryegrass (*Lolium perenne L*) dominated sward, which was subdivided into six paddocks. The sward was established four years before the experiment began and was subjected to strip-grazing management prior to the start of the experiment with treatments separated by electric fence.

The area to be grazed each day was adjusted by herbage allowance and pre-grazing HM. The pre- and post-grazing HM (kg DM/ha, above ground level) were estimated three times per week using a rising plate meter (Ashgrove Plate Meter, Hamilton, New Zealand). Each estimation considered 100 compressed sward height measurements by walking through the herbage in a ‘W’ pattern. Then, using a specific equation for autumn grassland of southern Chile [16], compressed height data (cm) was transformed into kg DM/ha. Post-grazing HM was estimated using the same methodology. The equation used is a s follows

Y=120X+350

R^2^ = 0.74

where Y is HM expressed in kg DM/ha, and X is average compressed height.

To create a difference of 1000 kg DM/ha between low and medium HM treatments, paddocks were grazed successively by non-experimental cows a month before the start of the experiment (April). Every time that herbage in the paddocks grew to 2000 kg DM/ha, 60% of each paddock was grazed by non-experimental cows and then used for L-AM and L-PM treatment groups. The remaining 40% of each paddock was grazed when herbage grew to 3000 kg DM/ha and then used by M-AM and M-PM treatments during the experiment.

### 2.3. Herbage and Supplement Sampling and Analyzes

Herbage samples were collected weekly at 10:00 hours for L-AM and M-AM treatments and at 17:00 hours for L-PM and M-PM treatments. All herbage samples were collected by cutting 4 cm above ground level. Supplement samples (grass silage and concentrate) were collected three times during the experiment and immediately frozen for chemical analysis. All samples were freeze-dried and, prior to chemical analysis, they were ground through a 1 mm screen (Willey Mill, Philadelphia, PA, USA) and analyzed for DM, CP, ADF, ash [17], NDF [18], and metabolizable energy (ME) [19,20]. 

### 2.4. Grazing Behavior

Grazing behavior was determined for each cow on two occasions during the experiment, on day 22 and 36 over 24 continuous hours. One trained observer was assigned to each group to record grazing behavior of each cows. Grazing activity was recorded using an instantaneous scan sampling at intervals of 10 minutes during daylight and 15 minutes during night [11,21]. Cows were considered as grazing when standing or walking with muzzle close to grass (i.e. head is below shoulders) [21] and eating activity was maintained for one minute or more. Cows were painted with large numbers on their sides to assist identification and avoid altering normal grazing behavior. All cows were observed outside of the daily electric fence. With the objective to evaluate the effect of treatments on cows grazing behavior through the day, the 24-h observations were divided into four time blocks (TB) as follows [22]: TB1: Time between AM milking to PM milking (07:00 and 14:00 h); TB2: Time between after PM milking to sunset (18:00 h); TB3: Time between sunset to 0:00; and TB4: Time between 0:00 to 06:45. Each time block represented a part of daily grazing time (minutes of grazing from total daily grazing time), multiplying the number of scan for 10 minutes for time block one and two, while TB3 and TB4 the number of scan was multiplied for 15 minutes.

### 2.5. Rumen Function

Ruminal concentration of NH_3_ was determined for each cow. Individual rumen samples were collected from three locations in the rumen (cranial, ventral, and caudal) at 08:00, 10:00, 13:00, 16:00, 20:00, 00:00, and 03:00 hours during days 15, 29, 43, and 57 of the experiment. Immediately after collection of rumen fluid, the samples (from each ruminal site) were bulked and a subsample of 10 ml was acidified with 0.2 mL of 50% trichloroacetic acid solution to measure rumen NH_3_ concentration by spectrophotometry (Spectronic Genesys 5^®^ spectrophotometer, Milton Roy, Ivyland, PA, USA), as described by Bal et al. [23]. 

### 2.6. Urine Sampling

Urine samples were collected immediately after rumen liquid samples. Approximately 40 mL of urine was collected via voluntary excretion or manual stimulation and then acidified with sulfuric acid (10% v/v) to minimize volatilization and then frozen for chemical analysis. Before chemical analysis, all samples were thawed and used to estimate N concentration (%) by a N autoanalyzer LECO FP528 based on DUMAS method [17]. Urinary urea (GLDH UV, HUMAN, Wiesbaden, Germany) was estimated using a Wiener Metrolab 2300 auto-analyzer (Wiener Lab., Rosario Argentina).

### 2.7. Statistical Analysis

Grazing behavior, urine N concentration, urine urea concentration, and rumen NH_3_ concentration were analyzed by repeated measurements ANOVA using the mixed model procedure (PROC MIXED; SAS, v9.4). The model included the fixed effects of treatment and period, random effect of cows, time of sampling (hour throughout day) as the repeated measurement, and the interaction between treatment and time of sampling. Compound symmetry was used as variance-covariance structure, because it showed the lower AIC value, therefore, it was the fitted model.

Chemical composition of pasture, herbage mass and sward height were analyzed using a mixed model procedure (PROC MIXED; SAS). The model included the fixed effects of treatment, day of sampling, and their interaction, and the random effect of paddock. 

Comparison between treatments was carried out with Tukey test. Results were considered significant at *p* < 0.05 and tendency at *p* < 0.1.

## 3. Results

Results for herbage and supplements chemical composition are presented in Table 1. Chemical composition of herbage was affected by treatments. The DM and WSC content of herbage were greater for M-PM and L-PM compared to L-AM (*p* < 0.05). Crude and soluble protein content of herbage were greater for L-AM compared to other treatments (*p* < 0.05). The WSC/CP ratio of herbage was greater (*p* < 0.05) for M-PM compared with L-AM and M-AM.

Results of HM and grazing behavior are presented in Table 2. Pre- and post-grazing HM were greater for M-AM and M-PM compared to L-AM and L-PM (*p* < 0.05). There was an interaction (*p* < 0.05) between treatment and time block for grazing time; Grazing time was longer for M-AM compared to other treatments at TB1 (*p* < 0.05). During TB2, the grazing time was longer for L-PM and M-PM compared to M-AM (*p* < 0.05). The grazing time at TB3 and TB4 was longer for M-PM and L-PM compared to L-AM and M-AM (*p* < 0.05).

**Figure 1:**
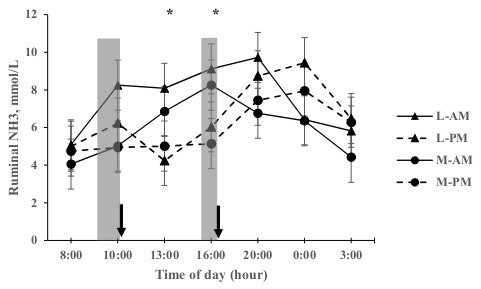
Diurnal variation of rumen ammonia concentration of dairy cows receiving a L-AM or L-PM and M-AM or M-PM at the Agricultural Research Station at the Austral University of Chile. ■: Grass Silage supplementation; **↓**: New fresh herbage allocation. * Indicate a significant difference between treatments (*p* < 0.05). Pasture was mainly dominated by *Lolium perenne L*.

Results of rumen NH_3_ concentration, urine urea, and urine N concentration are presented in Table 3. Rumen NH_3_ concentration was greater for L-AM compared to M-AM and M-PM (*p* < 0.05). When treatments were evaluated at different time throughout day (see Figure 1), greater rumen NH_3_ concentrations were observed at 13:00 and 16:00 h for L-AM compared to L-PM and M-PM (*p* < 0.05). There was a tendency for ruminal NH_3_ concentration to be greater for L-AM at 10:00 h (*p* = 0.06). In addition, a tendency (Figure 1; *p* = 0.08) for greater ruminal NH_3_ for M-AM was observed at 0:00 h.

**Figure 2:**
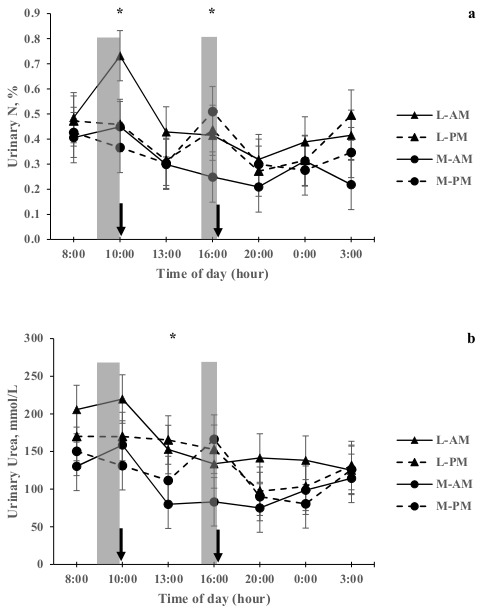
Diurnal variation of (**a**) urinary N and (**b**) urea excretion of dairy cows receiving a L-AM or L-PM and M-AM or M-PM at the Agricultural Research Station at the Austral University of Chile. ■: Grass Silage supplementation; **↓**: New fresh herbage allocation. * Indicate a significant difference between treatments (*p* < 0.05). Pasture was mainly dominated by *Lolium perenne L*.

There was a treatment effect (*p* < 0.05) on UN concentration with M-AM being lesser than L-AM (Table 2). Urinary urea concentration was lower (*p* < 0.05) for M-AM and M-PM compared to L-AM. When treatments were evaluated at different time throughout day, UN concentration was greater for L-AM than other treatments at 10:00 h (*p* < 0.05). In addition, UN concentration was greater for M-PM compared to M-AM at 16:00 h (*p* < 0.05). Urea concentration in urine was lower for M-AM compared to L-PM and L-AM at 13:00 h (*p* < 0.05). A tendency for greater urinary urea concentration was observed for L-AM at 08:00 h and 10:00 h (Figure 2; *p* = 0.08).

## 4. Discussion

This is the first empirical study supporting the modelling of Gregorini et al. [24] that simulates how changes in HM and timing of herbage allocation alter the diurnal pattern of UN.

### 4.1. Grazing Management and Grazing Behavior

Pre- and post-grazing HM were greater for treatments receiving a medium HM in the morning or afternoon compared to treatments receiving a low HM in the morning or afternoon. The mean HM for L-AM and L-PM was 1850 kg DM/ha, compared to 3030 for M-AM and M-PM (Table 2), indicative that the difference between low and medium HM was greater than the expected target difference (1000 kg DM/ha). However, this difference between low and medium HM was lower than reported by Pérez-Prieto et al. [10] and Wales et al. [25], where differences between low and high HM were 2300 and 1700 kg DM/ha, respectively. 

Total grazing time was lower for M-PM among treatments, in response to grazing time is reduced as pasture height increase, improving the facility for cows to graze the pasture [10,26]. Cows receiving a low or medium HM in the afternoon spent more time grazing in TB2, which was extended to TB3 (between afternoon milking to midnight), suggesting that cows concentrated the grazing activity during time of the day where pasture had a better nutritional value (Table 1). On the other hand, cows receiving a new HM spent more time grazing in TB1, e.g. between morning and afternoon milking, where pasture showed a lower nutritive value (Table 1). However, the L-AM and M-AM treatments spent more than 100 minutes grazing in TB2, which suggested that cows had the opportunity to grazed pasture with better nutritive value, especially for treatment receiving a medium HM in the morning.

### 4.2. Chemical Composition of Herbage

Chemical composition of herbage was modified by the combination of timing of herbage allocation and HM. In this way, the best chemical composition, in terms low CP content and high DM and WSC content, was observed for L-PM and M-PM compared to L-AM (Table 1). This can be explained by the diurnal variation of herbage chemical composition, with a high DM and WSC and low CP in the afternoon, in response to moisture loss, WSC accumulation, and dilution of CP concentration in the plants [27]. Several studies [11,12,28] support our results, where afternoon herbage had a greater DM and WSC and lower CP content than morning herbage. However, chemical composition of herbage was similar between M-AM and M-PM (Table 1), which can be attributed to a similar reduction in CP and increasing in WSC and DM content in the herbage as HM is increased [29], suggesting that low nutritive value of morning herbage can be improved by increasing HM (+ 1 ton/ha).

### 4.3. Urinary N and Urea Concentration and Rumen NH_3_

Urinary N and urea concentration were lower for M-AM compared to L-AM, but similar between M-AM and M-PM (Table 3). This can be related to changes in the daily eating pattern and its relationship with diurnal variation of herbage chemical composition. Considering that M-AM received a medium HM, it is possible that available HM in the afternoon was greater for this treatment compared to L-AM, where herbage has a greater DM and WSC and lower CP than morning herbage [30]. This result suggests that WSCP/CP and WSC intake for M-AM could be greater than reported here, which would be associated with the herbage sampling used in this experiment. Pasture sampling for M-AM were collected in the morning, therefore, nutrient intake reported were based on morning herbage samples, which does not consider the nutritional value of afternoon pasture. Additionally, the similar UN concentration and different total grazing time between M-AM and M-PM, suggests that grazing intensity during the day and diurnal variation in the chemical composition of herbage altered the UN concentration instead of total grazing time.

Our results show a greater pasture WSC intake for M-PM compared to M-AM (L-AM: 0.46 kg WSC/d/cow, L-PM: 0.62 kg WSC/d/cow, M-AM: 0.63 kg WSC/d/cow, and M-PM: 0.75 kg WSC/d/cow; unreported data), which does not consider the herbage chemical composition of M-PM at TB3, where the cows grazed for 30% of the available time, suggesting that herbage chemical composition between sunset and midnight (TB3) could have modified UN concentration in response to changes in the intake of N, WSC and water. In addition, the DM content in the morning herbage was 2.1% lower for M-AM than M-PM, which suggests a greater water intake for treatments receiving a new HM in the morning, diluting the negative effects of high herbage CP content in the morning and explaining the similar UN concentration with M-PM. It has been observed that slight reduction in the herbage DM content from 19% to 16% is enough to reduce the UN concentration from 5.5 g N/L to 3.7g N/L [7]. However, Cosgrove et al. (2017) did not find a difference in UN concentration between herbage with 13% DM (afternoon) and 12% DM (morning) under autumn conditions, which can be associated with the low difference between morning and afternoon herbage (+1%) compared to the current experiment (+2.1%) and that reported by Pacheco et al. [31] (+3%). 

The greater rumen NH_3_ for L-AM compared to L-PM and M-PM at 13:00 and 16:00 h (Figure 1) can be associated with the morning herbage allocation for M-AM and L-AM, where the rumen NH_3_ concentrations increased quickly during the first four hours once grazing commenced and then remained high for eight hours as reported as also reported by Trevaskis et al. [32] and Ueda et al. [33]. 

### 4.4. Relationships Between Daily Variation of Urinary N Excretion and Rumen NH_3_

Our results indicate that there were two times during the day when urinary urea and N concentration had a maximum peak (Figure 2a,b), being at 10:00 h for the L-AM treatment and at 16:00 h for M-PM (compared with M-AM). This suggests that simple changes in grazing management such as time of herbage allocation and HM modify the UN concentration pattern, supporting the model described by Gregorini et al. [34]. In this way, when cows received a daily supplement with high soluble protein (e.g. grass silage) during the normal 24-hour herbage allocation, the peak of UN concentration occurred two hours after grass silage allocation. These results are supported by Cosgrove et al. [35], who found greater UN concentration three hours after herbage allocation (high soluble protein) in grazing dairy cows. 

The difference in time to peak UN concentration following feeding reported here (two hours following silage supplement allocation) and by Cosgrove et al. (2017) (three hours following pasture allocation) could be attributed to the greater and faster release of non-protein N from grass silage into the rumen compared with herbage N, in response to decrease of WSC being replaced by fermentation products, reducing the energy supply for microbial growth [36]. Therefore, two hours after grass silage supplementation was enough to produce a substantial breakdown of silage protein, being converted into NH_3_ in the rumen [37], absorbed into the blood, converted into urea in the liver and finally excreted through the urine [38]. This result is supported by the tendency for greater ruminal NH_3_ concentration for L-AM at 10:00 h, indicative of high conversion of grass silage N into NH_3_ in the rumen and explaining the peak of urinary N and urea at this time. These results indicate that WSC intake from concentrate was not enough to stabilize ruminal NH_3_ levels [32] in L-AM at 10:00 h, suggesting that moderate supply of concentrate before herbage allocation may not be enough to reduce the peak of UN concentration. Hence a greater supply of highly-degradable fermentable carbohydrate supplementation could improve the N partitioning, especially at the beginning of grazing.

In addition, Betteridge et al. [39] reported a peak UN concentration 10 hours after herbage allocation, attributed with the digestion and metabolism of N, while Shepherd et al. [40] found a peak UN concentration at 15:30 and 21:30 h, which preceded bouts of grazing intake. The difference with the current experiment could be associated with different grazing management and supplementation; Betteridge et al. [41] delivered a new allocation of fresh herbage each morning without supplementation, while Shepherd et al. [40] moved cows off herbage to a stand-off pad for six hours per day in autumn. All management differences most likely triggered differences in nutrient flow through the day and eating behavior among experiments.

There was no relationship between ruminal peak of NH_3_ and UN concentration in the afternoon (Figure 1 and Figure 2a), suggesting that other factors are contributing to diurnal variation of UN at this time [35]. A possible hypothesis is that a long fasting period (herbage allowance offered once per day) could be limiting the rumen function and the use of urea-N recycling, suggesting that animals were unable to recycle the input N after a long fasting period, and the N was instead excreted through the urine. However, this would be a short-term response, being quickly reversed when the animals began to receive a constant flow of nutrients, which is reflected in the normal pattern of ruminal NH_3_ production after a new break of fresh herbage is allocated (10:00 and 16:00 h, see Figure 1). This is also supported by the lack of other urinary urea and N concentration peak (see Figure 2a,b) after a new herbage allocation, suggesting a better use of recycled urea-N. Thus, the WSC intake from concentrate after long fasting period was not enough to reduce the amount of recycled urea returning to the ornithine cycle. This is supported by the results of Huntington et al. [42], who observed that a high carbohydrate supplementation in forage fed steers decreased the return of urea-N to the ornithine cycle (as a proportion of recycled urea-N to the gastrointestinal tract). 

### 4.5. Practical Implications

Our work confirms that the combined effects of HM and time of herbage allocation mean cows consume herbage with different chemical composition (DM, CP, and WSC), leading to modification of eating behavior, daily pattern of rumen NH_3_ and daily pattern of urinary N concentration. In this way, it would be possible to reduce the amount of N excreted in the pasture by altering the traditional daily grazing management. Findings described above suggest maintaining the animals in the holding pen at the milking parlor for two hours after morning silage supplementation for L-AM and two hours after afternoon silage supplementation for M-PM, which could allow for the collection of the urine during the time of peak N concentration and thereby, returning the animals into the pasture at a time of day when urinary N concentration decreased.

## 5. Conclusions

Herbage mass and timing of herbage allocation could be used to alter the diurnal urea and N concentration pattern in response to changes in chemical composition of herbage, daily grazing pattern and thereby, ruminal NH_3_ concentration. The maximum peak urinary N concentration was observed at 10:00 h for L-AM, which was associated with an increased ruminal NH_3_ concentration. A second peak in urinary N concentration was observed at 16:00 h for M-PM, but this was not associated with ruminal NH_3_ concentration, indicating there are more, as yet unidentified, factors influencing this pattern.

## Figures and Tables

**Table 1:** Chemical composition of predominantly perennial ryegrass (*Lolium perenne L.*) pasture herbage and supplements (perennial ryegrass silage and concentrate fed to cows receiving a low herbage mass in the morning (L-AM) or afternoon (L-PM) and medium herbage mass in the morning (M-AM) or afternoon (M-PM) at the Agricultural Research Station at the Austral University of Chile.

	Treatments				SEM ^4^	*p*-Value	Supplements			
	L-AM	L-PM	M-AM	M-PM			Grass Silage	SEM	Concentrate ^1^	SEM
DM, %	11.5 b	14.3 a	12.1 ab	14.2 a	0.57	<0.01	37.3	2.75	86.4	0.08
CP, %	33.2 a	27.6 b	26.4 b	23.7 b	0.98	<0.01	14.9	1.35	11.5	0.56
SP, %	12.8 a	10.6 b	10.0 b	8.9 b	0.48	<0.01	-	-	-	-
NDF, %	49.5	48.1	50.5	48.1	1.92	0.77	46.6	1.28	32.3	1.29
ADF, %	21.8	21.6	23.4	23.3	0.52	0.06	28.9	0.45	15.5	0.91
ME ^2^	2.80	2.82	2.75	2.75	0.03	0.24	2.8	0.01	3.1	0.05
WSC, %	5.7 b	8.2 a	7.1 ab	8.8 a	0.24	<0.01	-	-	-	-
WSC/CP ^3^	0.18 c	0.3 ab	0.27 b	0.38 a	0.02	<0.01	-	-	-	-
pH	-	-	-	-	-	-	4.3	0.16	-	-
N-NH_3_, %	-	-	-	-	-	-	8.2	0.54	-	-

DM, Dry Matter; CP, Crude protein; SP, soluble protein; NDF, Neutral detergent fiber; ADF, acid detergent fiber; ME, ^2^ Metabolizable energy (Mcal ME/kg DM); and WSC, water soluble carbohydrates, N-NH_3_, ammoniacal nitrogen. Means within a row with different letters differ (*p* < 0.05). ^1^ Concentrate containing 49.3 corn, 11.5 soybean meal, 30.0 beet pulp, 4.6 beet molasses, and 4.5 mineral mix on a dry matter basis). ^3^ Water soluble carbohydrates/Crude protein ratio. ^4^ Standard error of the mean

**Table 2:** Grazing management and eating behavior of dairy cows receiving a L-AM or L-PM and M-AM or M-PM at the Agricultural Research Station at the Austral University of Chile.

Grazing Management and Behavior	Treatments				SEM ^2^	*p*-Value
	L-AM	L-PM	M-AM	M-PM		
Pre-grazing herbage mass	1853 b	1847 b	3078 a	2981 a	25.6	<0.01
Post-grazing herbage mass	1224 b	1198 b	1424 a	1424 a	7.69	<0.01
Grazing time ^1^, min						
TB1	210 b	105.5 c	243 a	64.5 d	6.8	<0.01
TB2	124 bc	151 a	106.5 c	139 ab	6.8	<0.01
TB3	41 b	111 a	38 b	98 a	6.8	<0.01
TB4	27 b	46 a	13 b	29 a	6.8	<0.01

^1^ TB1: After AM milking to PM milking (07:00 to 14:00 h); TB2: After PM milking to sunset (18:00 h); TB3: After sunset to 0:00 h; and TB4: 00:00 h to 06:45. Means within a row with different letters differ (*p* < 0.05). ^1^ Pasture was mainly dominated by *Lolium perenne L*. Each time block represented a part of daily grazing time (minutes of grazing from total daily grazing time), multiplying the number of scans for 10 minutes for time block one and two, while TB3 and TB4 the number of scan was multiplied for 15 minutes. ^2^ Standard error of the mean

**Table 3:** Daily variation of rumen ammonia concentration and urinary N and urea excretion of dairy cows receiving a L-AM or L-PM and M-AM or M-PM at the Agricultural Research Station at the Austral University of Chile.

Rumen Ammonia and Urinary N Excretion	Treatments ^1^				SEM ^2^	*p*-Value
	L-AM	L-PM	M-AM	M-PM		
Rumen Ammonia, mmol/L	7.50 a	6.60 ab	5.96 b	5.93 b	0.32	0.01
Urine Nitrogen, %	0.46 a	0.39 ab	0.28 b	0.36 ab	0.04	<0.01
Urea, mmol/L	161 a	141 ab	105 b	123 b	11.3	<0.01

Means within a row with different letters differ (*p* < 0.05). ^1^ Pasture was mainly dominated by *Lolium perenne L*. ^2^ Standard error of the mean
